# The Synaptic Theory of Memory: A Historical Survey and Reconciliation of Recent Opposition

**DOI:** 10.3389/fnsys.2018.00052

**Published:** 2018-10-26

**Authors:** Jesse J. Langille, Richard E. Brown

**Affiliations:** Department of Psychology and Neuroscience, Dalhousie University, Halifax, NS, Canada

**Keywords:** Hebb, memory, synaptic theory, molecular mechanisms, epigenetics, neurological disorders

## Abstract

Trettenbrein (2016) has argued that the concept of the synapse as the locus of memory is outdated and has made six critiques of this concept. In this article, we examine these six critiques and suggest that the current theories of the neurobiology of memory and the empirical data indicate that synaptic activation is the first step in a chain of cellular and biochemical events that lead to memories formed in cell assemblies and neural networks that rely on synaptic modification for their formation. These neural networks and their modified synaptic connections can account for the cognitive basis of learning and memory and for memory deterioration in neurological disorders. We first discuss Hebb’s (1949) theory that synaptic change and the formation of cell assemblies and phase sequences can link neurophysiology to cognitive processes. We then examine each of Trettenbrein’s (2016) critiques of the synaptic theory in light of Hebb’s theories and recent empirical data. We examine the biochemical basis of memory formation and the necessity of synaptic modification to form the neural networks underlying learning and memory. We then examine the use of Hebb’s theories of synaptic change and cell assemblies for integrating neurophysiological and cognitive conceptions of learning and memory. We conclude with an examination of the applications of the Hebb synapse and cell assembly theories to the study of the neuroscience of learning and memory, the development of computational models of memory and the construction of “intelligent” robots. We conclude that the synaptic theory of memory has not met its demise, but is essential to our understanding of the neural basis of memory, which has two components: synaptic plasticity and intrinsic plasticity.

## Introduction

When Pavlov first discovered classical conditioning, he was faced with the problem of how to interpret the behavior of his dogs. Some of his students and colleagues tried to interpret their behavior in terms of the subjective, introspective psychology of the day (Windholz, 1995). Pavlov, however, was determined to use an objective physiological interpretation and rejected subjective psychological terms. Once Pavlov’s lectures were translated into English (Pavlov, 1927, 1928a,b), Guthrie (1930) and Lashley (1930, 1931) published critiques which pointed out that Pavlov’s theories of brain and behavior ignored psychological concepts, such as attention and intelligence. Pavlov did not take these critiques lightly and wrote a lengthy reply (Pavlov, 1932). The Pavlov-Lashley debates regarding the neural basis of memory continued long after the death of Pavlov in 1936 (Lashley and Wade, 1946) and it was not until Hebb (1949) developed his neuropsychological theory that Pavlov’s physiological approach was integrated with the psychological concepts of perception and attention in the study of learning and memory.

Neurophysiological theories of synaptic plasticity, based on the experimental phenomena of long-term potentiation (LTP) and long-term depression (LTD), are now used to explain the neural basis of learning and memory (Nabavi et al., 2014; Yang et al., 2016). However, Trettenbrein (2016) has called into question the theory that synaptic change provides the neural basis for learning and memory, stating that: “Tentative evidence from a wide variety of work in neuroscience seems to provide support for the idea that the synapse is an ill fit when looking for the brain’s basic memory mechanism.” In this article, we examine Trettenbrein’s (2016) critiques of the synaptic theory of learning and memory and show that both theoretical and empirical research supports the concept that synaptic plasticity is an essential part of the complex cellular and molecular neurobiological changes which form the neural basis of learning and memory.

## Trettenbrein’s Critiques of the Neurophysiologic Explanation of Learning and Memory

Trettenbrein’s critique is that the synapse-centered view of learning and memory is ill-focused and that the neurobiological basis of learning and memory is still far from understood. There are six critiques of the synaptic plasticity theory of memory in Trettenbrein’s article, which will be addressed here: (1) the synapse may not be the sole locus of learning and memory; (2) a synaptic locus of memory does not fit well with philosophical and cognitive theories of learning and memory; (3) memories survive despite synapse destruction and synaptic and (or) protein turn-over; (4) evidence from spatial training suggests that there is a need to separate learning from memory; (5) existing learning mechanisms cannot explain information that is encoded in a single trial (Gallistel and Balsam, 2014); and (6) memory may be sub-cellular in nature. Trettenbrein (2016) makes the argument that the concept of the synapse as the locus of memory is not tenable and that a paradigm shift is necessary. However, he does not provide a new paradigm, except to suggest that “the memory mechanism is (sub-) molecular in nature” (page 3). The purpose of our article is to argue that there is a considerable literature on the neurobiology of learning and memory that shows the importance of synaptic plasticity as the first step in the chain of cellular and biochemical events involved in memory formation, and that, once memories are formed, synaptic modification is essential for their expression. We couch our discussion in terms of Hebb’s (1949) neuropsychological theory.

## Donald O. Hebb’s Theory of Learning and Memory

Hebb’s (1949) theory postulated that the neurophysiological changes underlying learning and memory occur in three stages: (1) synaptic changes; (2) formation of a “cell assembly”; and (3) formation of a “phase sequence,” which link the neurophysiological changes underlying learning and memory as studied by physiologists to the study of thought and “mind” as conceived by cognitive psychologists. As pointed out by Trettenbrein (2016), Hebb’s neurophysiological postulate (Hebb, 1949, page 62) states that:

“When an axon of cell A is near enough to excite a cell B and repeatedly or persistently takes part in firing it, some growth process or metabolic change takes place in one or both cells such that A’s efficiency, as one of the cells firing B, is increased.”

The “cell assembly” (Hebb, 1949, pages 69–74) is a set of neurons and the pathways connecting them, which act together, such that a stimulus activating pathway 1 will activate a reverberating circuit of N pathways (in Hebb’s example, *n* = 15). A cell assembly is a hypothetical reverberating system, proposed as a mediating process, an element of thought, capable of holding an excitation and thus of bridging a gap in time between stimulus and response (Hebb, 1972, pages 295 and 304). A series of cell assemblies, connected by neural activity over time is a “Phase Sequence,” which provides the neural basis for a “train of thought” from one cell assembly to another (Hebb, 1949, pages 79–106). The cell assembly thus “relates the individual nerve cell to psychological phenomenon” such that “a bridge has been thrown across the great gap between the details of neurophysiology and the molar conceptions of psychology” (Hebb, 1949, page 101). Hebb then elaborated on how this theory could account for learning and memory, how new learning could be associated with previous learning, and how “quick learning” (perhaps similar to the single trial learning of Gallistel and Balsam (2014)) might occur (Hebb, 1949, chapter 8). Hebb’s cell assembly theory thus showed how differences between psychologists and physiologists, who often use different definitions for the same phenomena, could be reconciled into a theory of the neurophysiological basis of learning and memory. It is important to note that Hebb’s postulate quoted above contains two concepts: synaptic plasticity and “some growth process or metabolic change” in the neuron, which has been termed “intrinsic plasticity” (Sehgal et al., 2013; Titley et al., 2017).

## Resolution of Recent Critiques Using Modern Neurophysiological Research

We believe that the critique of synaptic plasticity theory proposed by Trettenbrein (2016) can be resolved using Hebb’s synaptic theory, research based on cell assemblies as components of neural networks, and current research on the cellular and molecular basis of memory formation to indicate the essential nature of synaptic plasticity in understanding the neurobiology of learning and memory. The six critiques proposed by Trettenbrein (2016) will be addressed sequentially.

### The Synapse May Not be the Sole Locus of Learning and Memory

Trettenbrein (2016) describes the reservations held by some cognitive neuroscientists that the synapse is the “sole” locus of memory. While the synapse is an essential and highly studied component in the learning and memory process, it is not viewed by neurophysiologists as the sole locus of memory nor are its changes viewed as the sole basis of memory (Josselyn et al., 2015; Lisman et al., 2018). Memory is not represented by change at a single synapse, but by a series of processes involving molecular, biochemical, cellular and circuit level changes in widespread constellations of neurons throughout the brain. Specifically, when a strong stimulus occurs in the external environment, it drives high frequency stimulus trains (tetanic activity) in the neurons of a particular cell assembly, which, through their simultaneous synaptic activity, represent particular elements of the external stimulus (Buzsáki, 2010). Such a cell assembly or neural network has been identified for fear memory (Butler et al., 2015, 2018).

Propagation of strong excitatory currents through the synapses activate biochemical changes within neurons that lead to the strengthening of the synaptic connections within the circuit, or cell assembly. More precisely, *N*-methyl-D-aspartate receptor (NMDAR) induced calcium transients initiate intracellular signaling cascades leading to up-regulation of α-amino-3-hydroxy-5-methyl-4-isoxazolepropionic acid receptors (AMPARs), synaptic growth and phosphorylation mediated increases in AMPAR conductance at the post-synaptic compartment of synapses between cells participating in a given network (Lee et al., 2003; Bailey and Kandel, 2008; Henley and Wilkinson, 2013). Both pre-synaptic and post-synaptic changes are important in the strengthening of synapses between neurons comprising cell assemblies (Costa et al., 2017). Subsequent activity in the neurons of this cell assembly, with the connections between cells now demonstrating an increased synaptic efficacy, represents the elementary building blocks of learning, memory and other cognitive processes (Choi et al., 2018). Many of these cell assemblies firing consecutively form a phase sequence which connects the individual elements to produce a more complete mental representation, or train of thought (Almeida-Filho et al., 2014). Once the synaptic connections in the cell assemblies forming a phase sequence are modified by experience, subsequent activation of this specific array of neurons results in the experiential recollection of the memory stored in the constellation of modified synaptic connections (Nabavi et al., 2014; Josselyn et al., 2015; Butler et al., 2018).

Synaptic change is thus the first step in a series of events which link molecular activity at the synapse and the subsequent intracellular biochemical cascades and cellular changes to the cognitive aspects of memory. The neurobiological basis of memory exists as a series of synapse-specific molecular and biochemical changes, including *de novo* protein synthesis, phosphorylation, up-regulation of synaptic receptors and synaptic growth within and between cell assemblies which results in long-term changes to synaptic efficacy (Lee et al., 2003; Bailey and Kandel, 2008; Henley and Wilkinson, 2013; Jarome and Helmstetter, 2014). The synapse is the location where these biochemical changes are initially manifested and is altered through the intracellular changes and, therefore, is an integral part of the neurobiology of memory (Mayford et al., 2012). Any memories stored in the neurons of the cell assembly or phase sequence are expressed through synaptic modifications (Caroni et al., 2014; Khalaf and Gräff, 2016; Knoblauch and Sommer, 2016). Thus, the synapse is not the sole locus of memory nor is it the neurobiological basis of memory, but it is an integral component of the memory process. The locus of memory is the particular set of neurons comprising a cell assembly or phase sequence connected by synapses which are activated and modified by experience. Memory formation is the result of both synaptic plasticity and intracellular (intrinsic) plasticity (Titley et al., 2017; Lisman et al., 2018).

### A Synaptic Locus of Memory Does Not Fit Well With Philosophical and Cognitive Theories of Learning and Memory

Gallistel and Matzel (2014) and Trettenbrein (2016) stated that there was an incongruity between the theories of cognitive neuroscience and the idea of the synapse serving as the locus of memory. More precisely, he suggested that the synapse may be too complex to underlie a process as fundamental and essential as memory and that the elementary unit of memory is more likely a molecular or submolecular unit (Gallistel and King, 2009). As stated in “The Synapse May Not be the Sole Locus of Learning and Memory” section, memory formation has two components; synaptic changes within a cell assembly and intracellular biochemical changes. Memory involves many molecular and submolecular components, from the activation of second messenger systems, protein kinases and nuclear binding proteins to the transcription of DNA and translation of RNA, both locally at the synapses and in the nucleus and soma of the neurons (Fernandez-Moya et al., 2014; Jarome and Helmstetter, 2014). The resulting proteins and associated molecular changes serve as the neurobiological basis of memory, but the locus at which these sub-cellular changes are expressed is the synapse, itself a complex biochemical structure (Craig et al., 2006), indicating the important role the synapse and the network of synapses forming a cell assembly plays in both the formation and expression of memories in the neural tissue (Butler et al., 2015; Josselyn et al., 2015; Choi et al., 2018).

### Memories Survive Despite Synapse Destruction and Synaptic/Protein Turn-Over

An important critique proposed by Trettenbrein (2016) is that memories often persist despite synapse destruction and for durations outlasting the turnover of the synapse-modifying proteins which, according to synaptic theory, store memories. Many memories survive extreme brain remodeling such as occurs in insect metamorphosis, planarian brain regeneration, and mammalian hibernation (Blackiston et al., 2015). These seemingly incompatible findings are not at odds with a synaptic theory of memory. Hebb (1949; page 129) suggested that once a memory phase sequence has been formed from a set of cell assemblies, it becomes independent of any particular sensory stimulation; thus, a memory formed from visual stimuli may be activated by tactile or auditory stimuli. Experimental evidence shows that the synaptic weights of a predictable set of synapses are altered during learning and these modifications in synaptic weight are part of the processes underlying learning (Butler et al., 2015, 2018). Yet, subsequent destruction of the modified synapse rarely eliminates learning (Takeuchi et al., 2014; Trettenbrein, 2016). This is because a memory is not represented in a single modified synapse, but in a network of simultaneously activated cell assemblies and phase sequences connected by synaptic activity (Buzsáki, 2010). Memories are thus represented by networks of many synapses which are distributed widely throughout the brain and memory expression exists as a pattern of neural activity within a particular constellation of neurons (Josselyn et al., 2015).

Individual neurons are, as a consequence of their inputs, endowed with mnemonic receptive fields representing “bits” of information. Temporally contiguous or contingent stimuli cause coincidently active neurons (a particular constellation of which possess the mnemonic fields configuring to represent the stimulus) to have their connections strengthened via mechanisms of Hebbian spike-timing-dependent synaptic plasticity. Although numerous intracellular processes occur upstream it is this downstream, experience-dependent synaptic re-weighting which is ultimately responsible for the redirecting of information flow linking neurons, and thus the bits of information represented by their mnemonic fields, together into concepts (Hebbian cell assemblies) and associations (phase sequences). Thus, memory expression is an emergent network property resulting from distributed neural activity and destruction of a single synapse may weaken a memory, but does not cause the loss of an entire memory. Learning can produce persistent, sometimes even trans-generational (Lim and Brunet, 2013; Dias et al., 2015), epigenetic modifications as part of the memory formation process and these epigenetic marks may provide the basis of the intrinsic plasticity which allows memories to persist, in some capacity, following synapse destruction (Rajasethupathy et al., 2012; Khalaf and Gräff, 2016; Poo et al., 2016).

Synapses regularly turn over as do the proteins that strengthen these synapses during learning. How then can memories persist for years or even a lifetime, in the face of regular synapse and protein turnover? There are two, non-mutually exclusive, explanations for this. First, as discussed above, memory does not exist as a single modified synapse, therefore even the recycling and regular removal of certain connections allows a great many of the synapses in the cell assembly representing a memory to remain, allowing the memory, even if weakened, to persist. Attardo et al. (2015) found that dendritic spines were turned over on time scales comparable with those of systems level memory consolidation. Thus, the transience of individual dendritic spines in the hippocampus may serve to ensure this rapid acquisition system remains labile to encode future experience, and that information is not redundantly represented in the brain, once information has been shuttled to the neocortex. Neocortical dendritic spines have a heightened permanency relative to those in the hippocampus (Yang et al., 2009), suggesting that spine turnover is not a hindrance to the synaptic theory of memory. The second explanation focuses on the persistence of memory in the face of rapid protein turnover. Strong stimulation of a synapse (for example, by late-phase LTP, L-LTP) leads to the synthesis of an atypical protein kinase C isoform known as protein kinase M Zeta (PKMZ; Neves et al., 2008; Sacktor, 2008). PKMZ has been called the “memory molecule” as it maintains the molecular and biochemical alterations at the synapse laid down during learning, allowing the memory trace to persist despite continued protein turnover (Glanzman, 2013), providing the basis of Hebb’s “reverberating circuit.” For example, AMPAR up-regulation, a common synaptic modification during memory formation which promotes increased synaptic efficacy, is short-lived and is readily susceptible to down-regulation by endocytic internalization. PKMZ both prevents AMPAR endocytosis (Yao et al., 2008; Hardt et al., 2014) and ensures that AMPAR up-regulation continues, maintaining the elevated synaptic efficacy produced by prior learning (Migues et al., 2010). This allows the memory which persists, in part, through sustained biochemical changes at the synapse, to be carried forward in time for durations exceeding the turnover of individual macromolecules.

**Figure 1:**
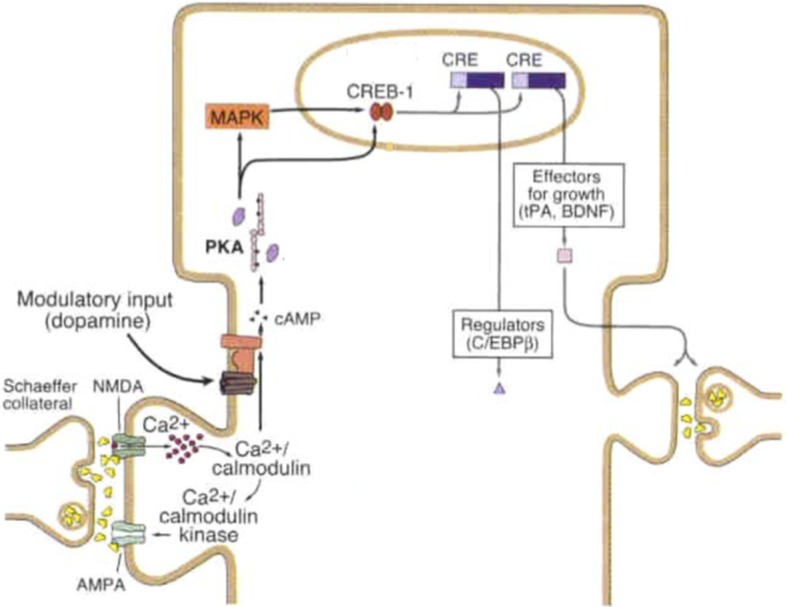
Late-long-term potentiation (L-LTP) mechanism at Schaeffer collateral synapse. Strong synaptic stimulation results in high magnitude calcium (Ca^2+^) influx, binding of calcium to calcium calmodulin which subsequently binds to calcium/calmodulin kinase and activates adenylyl cyclase (AC). AC generates the second messenger cyclic adenosine mono-phosphate (cAMP), cAMP activates PKA which translocates to the nucleus and activates mitogen activated protein kinase (MAPK) both of which turn on the transcriptional activator cAMP responsive element binding protein-1 (CREB-1). Upon CREB-1 binding to CRE transcription produces synapse modifying gene products including regulatory and growth proteins. Modulatory input, shown here as dopaminergic, can facilitate activation of AC and subsequent synaptic strengthening. Figure modified from Kandel (2001) License number 4363260804716.

But what happens when PKMZ, itself a protein susceptible to degradation, is broken down? The answer to this is what makes PKMZ a candidate for a “memory molecule.” PKMZ activity is self-sustaining since a continual positive feedback loop exists between the kinase activity and *de novo* PKMZ synthesis (Kwapis and Helmstetter, 2014). Once up-regulated, PKMZ molecules are autonomous and constitutively active owing to the activity of a catalytic domain and the lack of dependence on second messengers or auto-inhibition due to the absence of regulatory domains (Hernandez et al., 2003). In addition to its synaptic role PKMZ also acts in the nucleus to promote memory maintenance through epigenetic and transcriptional regulation (Ko et al., 2016). Mechanistically, the intracellular cascades activated during intense learning (LTP) drive the translocation of molecules, such as cyclic adenosine mono-phosphate (cAMP)-dependent protein kinase A and CaMKII, to the nucleus where they increase cAMP responsive element binding protein (CREB)-regulated CRE-driven transcription (Kandel, 2012) as shown in Figure 1, producing mRNA’s of memory-related proteins, including PKMZ (Lisman, 2017b). Although many of these PKMZ mRNAs bind RNA-binding proteins, such as staufen 1, and translocate to afferent neurites (Doyle and Kiebler, 2011), some are translated in free cytoplasmic ribosomes in the soma. These somatically translated PKMZ molecules then undergo importin-α mediated nuclear translocation where they phosphorylate CREB-binding protein (CBP). CBP regulates the accessibility to and transcription of proteins involved in synaptic potentiation and memory maintenance through its histone acetyltransferase and transcriptional co-activator activity, respectively (Ko et al., 2016). As long as PKMZ remains active and there is an absence of forces which terminate its activity (such as LTD), it will continue to sustain the biochemical changes at the synapse which serve as the neurobiological basis of memory, allowing the memory to persist for durations far exceeding the turnover of its component molecules. This mechanism may also underlie the stability of memories following brain remodeling (Blackiston et al., 2015). Additionally, memory persistence may be conferred, in part, by structural changes and growth at the synapse (Bailey et al., 2015), as structural changes last longer than many of the synaptic plasticity-related biochemical modifications (as discussed in “Synaptic Remodeling During Memory Formation” section). Thus, a synaptic theory of memory can account for the persistence of memories in spite of synaptic and protein turnover.

### Evidence From Spatial Training Suggests That There Is a Need to Separate Learning From Memory

Trettenbrein (2016) notes the need to separate learning from memory, citing literature showing that hippocampus-dependent spatial memory formation can occur even when NMDARs, which are thought to be necessary for LTP, are blocked (Saucier and Cain, 1995; Lüscher and Malenka, 2012). However, not all forms of hippocampus-dependent learning are NMDAR-dependent. For example, LTP in the hippocampal mossy-fiber pathway is NMDAR-independent (Johnston et al., 1992), yet LTP in this pathway is involved in spatial memory (Rekart et al., 2007). Thus, the involvement of the mossy fiber pathways in spatial memory offers an explanation for how learning can occur in the absence of NMDARs under certain conditions. NMDAR-independent synaptic potentiation is commonly categorized as a form of non-Hebbian synaptic plasticity. Kato et al. (2009) demonstrated that synaptic potentiation can occur in response to strong post-synaptic depolarization, without the requisite pre-synaptic activity seen in Hebbian models of plasticity. This form of synaptic plasticity is clearly NMDAR independent (as NMDARs require coincident pre- and post-synaptic activity) and instead utilizes post-synaptic voltage-dependent calcium channels. Additional forms of non-Hebbian learning are known to exist, including cerebellar learning which can result from a timing protocol opposite that of the spike-timing-dependent plasticity (STDP) originally postulated by Hebb (Hebb, 1949; Piochon et al., 2013). Even though cerebellar Purkinje cells can learn the interval timing involved in temporal memory traces, this does not rule out the synaptic theory of memory as the memory trace may be “coupled to a specific subset of synapses or dendritic compartments, but it is not exclusively reliant on a change in the strength of those synapses” (Jirenhed et al., 2017, page 6131). The problem with this argument is that even if “single Purkinje cells can learn response sequences” these cells rely on modified synaptic connections with other cells in order to express these memories. Therefore, non-Hebbian models of synaptic plasticity provide a mechanism by which learning can proceed, in certain cases, in the face of NMDAR blockade.

It is also difficult to separate learning from memory on theoretical grounds as learning and memory are inextricable parts of a continuum. Learning is the acquisition of new information or the modification of existing information, skills, etc., and that information must be represented in neural tissue, even if temporarily as occurs in sensory and working memory (Miller et al., 1960; Cowan, 2008; Bradley and Pearson, 2012). Synaptic plasticity provides a model for describing the continuity between learning and memory. The more incidental the learning (i.e., the less frequently the material being learned is rehearsed), the weaker and more transient the synaptic change by which that learning will be manifest, termed short-term potentiation (STP). On the other hand, more frequent or intense learning bouts will cause information to be stored in a series of persistent synaptic modifications, known as LTP. These stages (STP and LTP) are not discrete, binary states, but form a continuum (Volianskis and Jensen, 2003; Volianskis et al., 2013). Variability in learning intensity and frequency can cause activated synapses to become more or less potentiated, shifting memory strength and duration accordingly. This indicates that learning and memory are inextricable parts of a continuum, where the duration and strength of a memory is graded by the frequency and intensity of learning bouts and memory cannot occur without learning (Takeuchi et al., 2014), except in trans-generational epigenetic memories (Lim and Brunet, 2013; Dias et al., 2015). Therefore, it is not theoretically feasible to separate learning from memory as they are inextricable parts of a continuum, as discussed in “Existing Learning Mechanisms Cannot Explain Information That is Encoded in a Single Trial” and “Memory May Be Sub-Cellular in Nature” sections.

### Existing Learning Mechanisms Cannot Explain Information That Is Encoded in a Single Trial

Traditional approaches to associative learning suggest that the number of trials and the CS-US interval are the primary determinants of learning (Hawkins et al., 2006), supporting Hebb (1949) principle that the repeated, simultaneous activation of a synapse leads to the strengthening of that synapse. Gallistel and Balsam (2014), cited in Trettenbrein (2016), suggest that it is physiologically difficult to explain how synaptic learning mechanisms can encode information after a single trial. However, one trial inhibitory avoidance conditioning induces LTP in the CA1 neurons of the hippocampus (Whitlock et al., 2006) and a single exposure to a novel environment can stimulate LTP and activate the immediate-early gene *Arc* in CA3 neurons of the hippocampus (Miyashita et al., 2009). Novel stimuli also stimulate the release of neuromodulatory transmitters such as dopamine and acetylcholine which contribute to the synaptic modifications underlying one trial learning (Takeuchi et al., 2016). Therefore, a theory which regards the synapse as integral to memory formation can address this criticism. Bissière et al. (2003) and Marowsky et al. (2005) have shown that dopamine, acting as a neuromodulator, facilitates Hebbian LTP in the lateral amygdala during aversive learning. Furthermore, Bush et al. (2010) describe the ability of beta-noradrenergic receptors (β-ARs) to modulate threat learning, perhaps through influencing STDP (Pawlak et al., 2010). Johansen et al. (2014) demonstrated that in learning following very few trials, Hebbian plasticity mechanisms alone were insufficient to produce plastic changes in the lateral amygdala. Learning successfully occurred, however, when these Hebbian plasticity mechanisms were paired with the co-activation of β-ARs, emphasizing the importance of neuromodulation in this form of learning. Theoretically, while a single small shock may produce STP (early E-LTP), a larger shock and the concomitant neuromodulation, will result in a more sustained potentiation (long L-LTP). In sum, neuromodulation and the activation of immediate early genes, working in concert with Hebbian plasticity, provide a mechanistic explanation for how instances of single trial learning are explained by a synaptic theory of memory.

### Memory May Be Sub-cellular in Nature

Trettenbrein’s (2016), critique of synaptic theory and in support of a submolecular basis for memory, quotes from Gallistel and King (2009, page 282) that “our skepticism rests most strongly on the fact that the synapse is a circuit-level structure, a structure that it takes two different neurons and a great many molecules to realize. It seems to us likely for a variety of reasons that the elementary unit in the memory mechanism will prove to be a molecular or sub-molecular structural unit.” This statement contains two points, which need to be addressed independently. First, the fact that the synapse is a structure involving neuronal connections is not a burden to a synaptic theory of memory but an asset. Associative memories are formed through a rewiring of brain circuits (Fuster, 1997; Chau et al., 2014; Van Ooyen and Butz-Ostendorf, 2017) such that the collection of neurons (cell assemblies) representing stimulus “A” and those representing stimulus “B” are either connected by *de novo* synaptogenesis (Le Bé and Markram, 2006; Kwon and Sabatini, 2011); have existing synapses between these neurons potentiated; or a combination of both (Choi et al., 2018). Subsequent activity in “A” encoding neurons can then, through spreading activation, elicit activity in the neurons encoding “B” thus providing a neural link between the cell assemblies (a phase sequence) representing each of these two originally discrete stimuli (Anderson, 1983). As discussed in “Evidence From Spatial Training Suggests That There Is a Need to Separate Learning From Memory” section, learning and memory occur along a continuum, so the fact that synaptic encoding of a memory requires “a great many molecules to realize” is a benefit as it allows for the gradation of synaptic, and in turn memory, strength along a continuum in accordance with the salience of the information, or effortfulness of original learning (Liu et al., 2017). Furthermore, the biochemical and structural complexity of the glutamatergic synapse, actualized by these “great many molecules,” allows for several distinct forms of plasticity: (i) STP; (ii) early or late LTP (E-LTP or L-LTP); (iii) LTD; (iv) synaptic scaling; and (v) distance-dependent scaling (Volianskis et al., 2013; Lisman, 2017a). This diversity helps to explain how various types, durations and strengths of memory can be represented in the brain. So, the nature of the synapse as a circuit-level structure and the number of molecules involved in producing synaptic change are not hindrances to a synaptic theory of memory but strengths.

Second, considerable evidence exists for the intracellular chain of biochemical events, which lead to memory formation. As described by Kandel (Kandel, 2001; Kandel et al., 2014) and others (Guzman-Karlsson et al., 2014), synaptic activity triggers intracellular second messengers such as cyclic AMP, protein kinases, MEK-ERK pathways, CAMKII, CREB and binding to DNA for transcription of genetic information to mRNA and translation of this information for new protein synthesis; an overview of elements common to synaptic potentiation pathways is included as Figure 1. These intracellular pathways modulate synaptic activity by inducing changes in the quantity (Fleming and England, 2010) and conformational status of AMPARs (Lee et al., 2000); the surface area of the post-synaptic density, and in turn the size of the axon-spine interface (Desmond and Levy, 1986, 1988); the volume of the dendritic spine head (Matsuzaki et al., 2004; Bozdagi et al., 2010); and the growth of new synapses (Kandel, 2001). In addition, retrograde signals, such as nitric oxide (Padamsey and Emptage, 2013), are generated post-synaptically and travel to the presynaptic compartment where they elicit alterations as part of the potentiation process. These presynaptic alterations act to alter the probability and latency of presynaptic neurotransmitter release, modulate the magnitude of quantal release (Larkman et al., 1992; Sola et al., 2004; Enoki et al., 2009), the size of the pool of readily available synaptic vesicles, the quantity and responsiveness of presynaptic calcium channels and the set point of presynaptic calcium sensors (Kaeser and Regehr, 2014; Costa et al., 2017). These presynaptic modifications can act bi-directionally, to potentiate or depress synaptic efficacy (Fioravante and Regehr, 2011; Regehr, 2012; Yang and Calakos, 2013). Finally, CREB-mediated intrinsic plasticity can be described as being submolecular and has recently been implicated in processes of memory allocation, linking and integration (Josselyn and Frankland, 2018; Lisman et al., 2018; Sehgal et al., 2018). Thus, the suggestion put forth in Trettenbrein (2016) that memory is submolecular in nature is correct, but incomplete.

An additional piece of evidence supporting a synaptic plasticity model of memory is that both memory and synaptic plasticity consist of at least two discrete temporal stages. Short-term memory (using the neurophysiological definition) lasts for ~1–2 h. E-LTP, also, lasts for ~1–2 h and results from temporary modifications of pre-existing proteins which are sustained for as long as kinase activity (e.g., CaMKII) exerts dominance over phosphatase activity. Once dominance has shifted towards phosphatase activity, the biochemical substrates underlying the memory are rapidly removed and the memory is lost (Huang, 1998; Genoux et al., 2002; Munton et al., 2004). Long-term memory duration varies but is generally considered to persist for longer than 2 h and can last for days, weeks, years or even a lifetime. L-LTP, not coincidentally, lasts for durations comparable to those described for long-term memory (Costa-Mattioli et al., 2008). The protein synthesis underlying L-LTP is responsible not only for generating a greater number of synaptic potentiation promoting proteins and structural components but also for generating the persistent kinases capable of allowing these long-term memories to be maintained for such impressive durations (Jalil et al., 2015). Thus, this temporal overlap serves as evidence that the synaptic change occurring during E-LTP serves as the neurobiological correlate of short-term memory formation while those synaptic changes involved in L-LTP represent the neurobiological correlate of long-term memory formation.

A final component of Trettenbrein (2016) and Gallistel and King’s (2009) argument that memories might be submolecular is that, “neural computation is demonstrably incredibly fast, therefore making it much more likely that the memory mechanism is (sub-) molecular in nature so that computational machinery and memory can be located in close physical proximity in order to minimize the distance over which a signal has to be transmitted.” If it is assumed that by “computation” the authors are referring to the ability of the organism to take in, process and utilize information, then indeed memory is fast, but this rapidity can be explained while maintaining adherence to a synaptic theory of memory. The intracellular signaling cascades, protein synthesis and morphological changes that represent memory in a synaptic theory are indeed slow to occur, but one need not wait for these changes in order to utilize the memory. Memory is initially encoded in the mnemonic fields of a particular constellation of prefrontal cortical neurons as a reverberatory pattern of activity (Lorente De Nó, 1933; Hebb, 1949; Funahashi et al., 1989; Wang, 2005; Lara and Wallis, 2015). These electrical reverberations induce synaptic E-LTP which lays down information as a series of temporary, transcription independent, kinase activity-dependent, biochemical alterations (Huang, 1998) capable of maintaining information in the brain’s connectivity until the more permanent, transcription-dependent L-LTP-dependent processes can better establish this memory in the neural tissue (Vickers and Wyllie, 2007). Thus, a synaptic theory of memory ensures that the information is rapidly available for immediate computational usage, despite the temporal void that exists between the acquisition of this information and its later stabilization through the longer lasting *de novo* protein synthesis and morphological changes involved in consolidation. Once again, this is evidence that one need not stray from a synaptic theory in order to explain the complexities of memory.

Memory formation involves both genetic and epigenetic processes. The transcription of genetic information from DNA to mRNA involves histone acetylation, histone methylation and DNA methylation (Mazzio and Soliman, 2012; Zovkic and Sweatt, 2013). We believe these intracellular changes highlighted by Trettenbrein (2016) while implicated in memory formation and sustenance cannot provide a synapse independent explanation of memory. Such changes lack a means of modifying the flow of electrical activity and thus information in the brain necessary for the expression of experience-dependent memory. These changes instead regulate the expression of various genes the products of which re-weight synapses allowing a sustained redirecting of neural activity. The laying down of these epigenetic marks during learning and the influence that they then exert on both Hebbian and non-Hebbian synaptic plasticity has become a fruitful area of inquiry (Guzman-Karlsson et al., 2014; Kim and Kaang, 2017). These epigenetic processes allow modulation of memories by other chemical messengers, including neurotransmitters (dopamine, serotonin, etc.), hormones (corticosterone, androgens), neuropeptides (oxytocin, ACTH), and even cytokines (IL-1, IL-6, TNFα, etc.; Song et al., 2003; Hunter, 2012; Rajasethupathy et al., 2012; Trollope et al., 2012; del Rey et al., 2013; Tuesta and Zhang, 2014; Reizel et al., 2015; Haas et al., 2016; Madej et al., 2017; Prieto and Cotman, 2017), all of which can be activated by external and internal stimuli, including emotions, motivations, thoughts and memories (Davis et al., 1994; Berger et al., 2009).

In addition, the reactivation of stored memories causes them to become transiently deconsolidated and labile, providing an opportunity to amend preexisting memories with new information. These retrieved memories are then reconsolidated, leading to the confounding of original memories with new information and the storing of false memories (McKenzie and Eichenbaum, 2011; Ramirez et al., 2013; Almeida-Correa and Amaral, 2014). When a memory is explicitly retrieved, it is done so by driving reverberatory activity through the experientially modified synapses in the cell assemblies and phase sequences within which the memory is represented. This subjects it to the operations of working memory where it is Fragile and susceptible to alteration (Lee et al., 2017). The activated synapses produce intracellular biochemical changes and protein synthesis, as discussed in “Synaptic Remodeling During Memory Formation” section, which remodel the synapse in a manner consistent with the pattern of activity experienced at the synapse (i.e., according to the principles of STDP; Lee et al., 2011). The remodeling of the synapses in the cell assemblies and phase sequences underlying a memory can incorporate new information, or change the existing information, comprising the retrieved memory (Bonin and De Koninck, 2015; Hu and Schacher, 2015; Kastner et al., 2016). Thus, the neurophysiological basis of reconsolidation provides support for a synaptic theory of memory.

### Synaptic Remodeling During Memory Formation

As stated by Hebb (1949), “some growth process or metabolic change” takes place to increase the efficacy of cell A on the firing of cell B. The activation of intracellular biochemical pathways, gene expression, epigenetic factors, and protein synthesis leads to the remodeling (strengthening or weakening) of synapses in cell assemblies. There are many different types of growth processes and/or metabolic changes that lead to changes in synaptic strength, including: (1) excitatory; (2) inhibitory; (3) size of the synapse; (4) ion channels; (5) dendrites, boutons, et cetera (De Roo et al., 2008; Caroni et al., 2014; Bailey et al., 2015; Yang et al., 2016; Choi et al., 2018). Distinct activity patterns drive specific intracellular biochemical signaling cascades which serve to adaptively remodel the synapse during activity-dependent synaptic plasticity (Colicos, 2009, pages 50–52). As an example, adaptive, biologically relevant information which needs to be remembered in order to optimally organize behavior and cognition at a later time point is likely to drive tetanic activity through a particular set of synapses, producing large post-synaptic calcium transients. Calcium then binds to calcium binding proteins, including calmodulin, which activates various effectors including nNOS, CaMKII, post-synaptic adenylyl cyclase (AC), etc. These effectors then activate downstream kinases, genes and other proteins capable of remodeling the pre- and post-synaptic elements in such a way to confer an increased synaptic efficacy (Wong et al., 1999; Kandel, 2012; Choi et al., 2018). These remodeling changes, including the growth of pre-existing and the formation of new dendritic spines are correlated in magnitude with the degree of training (Xu et al., 2009; Yang et al., 2009). Thus, intracellular metabolic changes function to modulate synaptic remodeling and growth processes during memory formation.

## Reconciliation of the Differences Between Physiologists and Psychologists About the Role of Synaptic Plasticity in Learning and Memory

Our critique of Trettenbrein (2016) has focused on the term “demise” of the synaptic theory of memory. We believe that the synaptic theory of memory has not died but that there are two components of this theory: synaptic plasticity and intra-cellular biochemical changes. At issue is whether “memory” consists of the synaptic changes activated by intracellular biochemical changes OR whether memory consists of the intracellular biochemical changes expressed via synaptic plasticity. Our argument is that memory, as conceived by Hebb, consists inseparably of both synaptic plasticity and “intrinsic plasticity” of the neurons (Sehgal et al., 2013; Titley et al., 2017; Lisman et al., 2018).

### Synaptic Change and the Formation of Cell Assemblies Are Fundamental for Theories of Memory

Hebb (1949, 1959) realized that his theory would need revision in the light of new discoveries, but the fact that his ideas on synaptic plasticity (Favero et al., 2014), cell assemblies (Lansner, 2009; Wallace and Kerr, 2010) and phase sequences (Almeida-Filho et al., 2014) continue to stimulate new research and discussion is a tribute to his prescience. For example, Hebb’s ideas continue to stimulate new research on the physiological mechanisms of learning and memory (Schrader et al., 2002; Lisman et al., 2011; Johansen et al., 2014), learning and development (Munakata and Pfaffly, 2004), memory span (Oberauer et al., 2015), decision making (Wang, 2012) and language learning (Wennekers et al., 2006). Posner and Rothbart (2004, 2007) went as far as to suggest that Hebb’s ideas should be used to integrate the disparate branches of Psychology and Neuroscience.

### Cell Assemblies Have Been Verified by Neuroimaging

Hebb’s theories on the neurophysiological basis of learning and memory integrate synaptic neurophysiology with psychological concepts such as attention, perception, thought and mind—the concepts which Pavlov avoided in his objective approach to memory. Hebb’s theory effectively integrated Pavlov’s concepts of the physiology of learning with Lashley’s (1932) criticism that Pavlov ignored psychological concepts. Neuroimaging studies have shown the usefulness of Hebb’s ideas for understanding both the psychological and physiological mechanisms of memory. Memory processes have been shown by fMRI and other neuroimaging methods to be distributed across many cortical areas (Miyamoto et al., 2014). For example, Christophel et al. (2017) showed that different cortical neural networks are activated in different types of working memory, and O’Neil et al. (2012) found that different cortical regions were activated in recognition memory.

### Hebb’s Synaptic Learning Rule and Cell Assembly Theory Is Used in Computational Neuroscience and Robotics

Hebb’s concept of cell assemblies and phase sequences have been used to develop theories of the cortical control of behavior (Palm et al., 2014), network theories of memory (Fuster, 1997) and computer models of memory processes (Lansner, 2009). According to Palm et al. (2014; page 560), “the further development of cell assembly theory was mainly driven by neurophysiological and biophysical findings concerning the basic neuronal mechanisms and the detailed temporal processes of neuronal activation and interaction on one hand and by computational arguments and requirements on the other.” Cell assembly theory has resulted in the development of anatomical features that underlie the location of memory storage in the cortex (see Palm et al., 2014). Computer models of the brain use Hebbian learning rules and cell assemblies (Wennekers, 2007) to build neural networks based on spike timing dependent plasticity (Markram et al., 2011). The role of Hebbian theory in neural network modeling will continue to grow in importance (Buzsáki, 2010; Fotouhi et al., 2015). Furthermore, Hebbian learning rules and cell assemblies are now used in robotics (Wang et al., 2009; Calderon et al., 2013) and it is now possible for Hebbian learning rules to control brain-robot interfaces in neurorehabilitation (Gomez-Rodriguez et al., 2011; Takeuchi and Izumi, 2015).

### Abnormalities in Synaptic Plasticity Underlie Cognitive and Motor Dysfunctions Pain Mechanisms and Drug Addiction

The activation of the network of synaptic connections in a cell assembly requires changes in synaptic strength to establish the connectivity of the neurons in the cell assembly. Cell assemblies are essentially a collection of activated synapses and the sufficiently strong activation of these synapses causes biochemical changes in the neurons of the cell assembly. Thus, biochemical changes and gene activation within the neurons of a cell assembly are required to maintain memories (Li et al., 2016). These involve complex interactions between excitatory and inhibitory synapses (see Barron et al., 2017). The biochemical changes in the neurons of a cell assembly that are activated by transient changes in synaptic activity involve epigenetic mechanisms including chromatin remodeling which drives changes in the transcription and translation of information in the DNA, protein synthesis and cellular changes underlying learning and memory formation (Vogel-Ciernia and Wood, 2014). As stated by Hebb in 1949, the synaptic changes following repeated stimulation at a synapse lead to “some growth process or metabolic change… in one or both cells such that A’s efficiency, as one of the cells firing B, is increased.” Much of the neuroscientific research on the cellular and molecular basis of memory in the last 70 years has been oriented to finding these growth processes and metabolic changes that underlie memory (Josselyn et al., 2015; Poo et al., 2016). Synaptic change is not limited to learning and memory, but forms the basis of neural changes in perception (Dan and Poo, 2006; Yang et al., 2011), pain (Luo et al., 2014) and drug addiction (Jones and Bonci, 2005; Kauer and Malenka, 2007; Lüscher, 2013).

Neurological disorders which involve cognitive or motor dysfunction are the result of synaptic abnormalities (Amorim et al., 2015; Kouroupi et al., 2017). Synaptic dysfunction underlies neurodevelopmental disorders such as autism, Rett syndrome, Down syndrome and ADHD (Pettem et al., 2013; Moretto et al., 2018) and a wide range of neurological disorders of adulthood and aging, including Alzheimer disease, Parkinson’s disease, Huntington’s disease and multiple sclerosis (Henstridge et al., 2016; Rosales-Reynoso et al., 2016; Forner et al., 2017; Torres et al., 2017). For example, tau tangles and beta-amyloid peptide are elevated, intra- and extracellularly respectively, in the brains of Alzheimer’s disease patients and are known to impair synaptic function and cause synapse loss (Forner et al., 2017). Specifically, the accumulation of beta-amyloid oligomers in Alzheimer’s disease impairs hippocampal LTP and memory consolidation (Figueiredo et al., 2013; Bilousova et al., 2016; Yang et al., 2017) in addition to causing reductions in dendritic spine density, AMPAR numbers, synaptic strength (Rodrigues et al., 2016) and synapse loss (Sheng et al., 2012). Impaired hippocampal LTP and consolidation may explain the difficultly in forming new, lasting memories (Weintraub et al., 2012), termed anterograde amnesia, while decreases in synaptic strength (and thus removal of the physical substrates of memories) and synapse loss may explain the erasure of past memories in retrograde amnesia, both of which are characteristic of Alzheimer’s disease (Beatty et al., 1988). Thus, a synaptic plasticity theory of memory can explain the memory impairments in neuropathologic conditions such as Alzheimer’s disease.

### Contrary Opinions

The theory that synaptic connectivity forms the basis of cognitive functions is not universally accepted. In addition to Trettenbrein’s (2016) critique, models of learning and memory based on non-synaptic plasticity have been proposed (Mozzachiodi and Byrne, 2010; Cacha et al., 2017), but these appear to involve neuromodulatory actions which alter the parameters of LTP and LTD, and thus the efficacy of synaptic plasticity. In addition, Arshavsky (2006, 2017), Chen et al. (2014) and Bèdècarrats et al. (2018) have advocated a genomic rather than a synaptic theory of memory and argue that changes in individual neurons rather than interconnected neural networks form the basis of memory. Bèdècarrats et al. (2018) demonstrated that the transfer of RNA from trained to naïve animals is sufficient to induce behavioral correlates of memory and that these changes are dependent on DNA methylation. However, the modified expression of these gene products likely serves to change synaptic weights, an idea supported by Bèdècarrats et al.’s (2018) demonstration of an increase in sensory neuron excitability following RNA injection. While these genetic and epigenetic views are minority opinions, they cannot be ruled out. However, if cognitive processes reside in individual neurons or through genomic and epigenomic modifications, the only way that these cells can communicate with other neurons is through synaptic activity. Thus, even if one was to concede that memory may reside in individual cells or in genomic and epigenomic modifications, these cells form a cell assembly and the expression of that memory requires the activation of the synapses linking the cells. In order to facilitate the formation of a cell assembly, synaptic activation occurs and as a memory is consolidated into a cell assembly, synaptic modifications occur. Without these synaptic modifications, memories could not be expressed.

## Summary and Conclusions

Trettenbrein’s (2016) critiques of the synaptic theory of memory can be answered by the published evidence for the synaptic theory of memory, as argued in this article. The synaptic theory of memory remains the most empirically plausible explanation for the neurobiological basis of memory, even if it may need modification (Jirenhed et al., 2017). In this article we have addressed the six critiques raised by Trettenbrein (2016) and through the use of recent neurophysiologic literature, we have demonstrated that synaptic change is only the first step in formation of the cell assemblies and phase sequences postulated by Hebb (1949), which together constitute the Hebbian account of memory. We have shown how the concepts of synaptic change and the cell assembly are used to understand the neuroanatomical, cellular, molecular and genetic bases of memory and of neurological disorders. We have also shown how neuroimaging studies, computer modeling and robotics have used the Hebbian learning rules and cell assemblies to develop computer learning and “intelligent” robots. Our critique of Trettenbrein (2016) has focused on the term “demise” (death, downfall, disappearance or final fate) of the synaptic theory of memory. We believe that the synaptic theory of memory has not died, but has gone from strength to strength. But there are two components of this theory: synaptic plasticity and intra-cellular biochemical changes. At issue is whether “memory” consists of the synaptic changes activated by intracellular biochemical changes OR whether memory consists of the intracellular biochemical changes expressed via synaptic plasticity. Our argument is that memory, as conceived by Hebb, consists of both synaptic plasticity and “intrinsic plasticity” of the neurons (Sehgal et al., 2013; Titley et al., 2017; Lisman et al., 2018). You cannot separate one from the other.

## Author Contributions

JL and RB wrote the article. RB edited and supervised the project.

## Conflict of Interest Statement

The authors declare that the research was conducted in the absence of any commercial or financial relationships that could be construed as a potential conflict of interest.
